# Urban sanitation coverage and environmental fecal contamination: Links between the household and public environments of Accra, Ghana

**DOI:** 10.1371/journal.pone.0199304

**Published:** 2018-07-03

**Authors:** David M. Berendes, Amy E. Kirby, Julie A. Clennon, Chantal Agbemabiese, Joseph A. Ampofo, George E. Armah, Kelly K. Baker, Pengbo Liu, Heather E. Reese, Katharine A. Robb, Nii Wellington, Habib Yakubu, Christine L. Moe

**Affiliations:** 1 Department of Environmental Health, Rollins School of Public Health, Emory University, Atlanta, GA, United States of America; 2 Center for Global Safe Water, Sanitation, and Hygiene, Emory University, Atlanta, GA, United States of America; 3 Department of Biostatistics and Bioinformatics, Rollins School of Public Health, Emory University, Atlanta, GA, United States of America; 4 Noguchi Memorial Institute for Medical Research, University of Ghana, Legon, Accra, Ghana; 5 Council for Scientific and Industrial Research, Water Research Institute, Accra, Ghana; 6 TREND Group, Accra, Ghana; Johns Hopkins Bloomberg School of Public Health, UNITED STATES

## Abstract

Exposure to fecal contamination in public areas, especially in dense, urban environments, may significantly contribute to enteric infection risk. This study examined associations between sanitation and fecal contamination in public environments in four low-income neighborhoods in Accra, Ghana. Soil (n = 72) and open drain (n = 90) samples were tested for *E*. *coli*, adenovirus, and norovirus. Sanitation facilities in surveyed households (n = 793) were categorized by onsite fecal sludge containment (“contained” vs. “uncontained”) using previous Joint Monitoring Program infrastructure guidelines. Most sanitation facilities were shared by multiple households. Associations between spatial clustering of household sanitation coverage and fecal contamination were examined, controlling for neighborhood and population density (measured as enumeration areas in the 2010 census and spatially matched to sample locations). *E*. *coli* concentrations in drains within 50m of clusters of contained household sanitation were more than 3 log-units lower than those outside of clusters. Further, although results were not always statistically significant, *E*. *coli* concentrations in drains showed consistent trends with household sanitation coverage clusters: concentrations were lower in or near clusters of high coverage of household sanitation facilities—especially contained facilities—and vice versa. Virus detection in drains and *E*. *coli* concentrations in soil were not significantly associated with clustering of any type of household sanitation and did not exhibit consistent trends. Population density alone was not significantly associated with any of the fecal contamination outcomes by itself and was a significant, yet inconsistent, effect modifier of the association between sanitation clusters and *E*. *coli* concentrations. These findings suggest clustering of contained household sanitation, even when shared, may be associated with lower levels of fecal contamination within drains in the immediate public domain. Further research is needed to better quantify these relationships and examine impacts on health.

## Introduction

An estimated 68% of the world’s population live with improved sanitation (currently “basic” or “safely-managed” sanitation), classified by the Joint Monitoring Program (JMP) as systems that ensure the safe containment and separation of excreta from human contact onsite (though not necessarily along the entire sanitation chain, which is reserved for only “safely-managed” sanitation), thereby reducing health risk [1]. For the 32% without improved sanitation, this health risk can stem from exposure to environmental fecal contamination in the household or public domain (which we classify according to Cairncross et al. [2] as areas outside of the household’s control—often outside the formal household compound—including streets, public drains, and other public areas), especially in urban areas with high population density [3–5]. Systems-level approaches to the entire sanitation chain need to be considered to contain excreta across private and public domains, as reflected in Sustainable Development Goal (SDG) 6.2 [6–9].

In low-income countries, improved sanitation infrastructure and services have not kept up with population growth in urban areas. From 1990 to 2015, urban sanitation coverage only increased from 79% to 82% worldwide, and from 37% to 47% in the world’s poorest countries [9]. Meanwhile, over half of the world’s population currently live in urban areas, and this proportion is expected to increase to two-thirds of the world’s population by 2050 [10]. Growth in low-income urban neighborhoods in poor countries is expected to parallel overall urban growth: doubling from 2001 to 2030, adding to the 600 million urban dwellers without access to sanitation [6,11,12].

The health risks associated with poor urban sanitation are complex because exposures to fecal contamination occur both inside and outside the household [2]. While numerous studies have linked poor urban sanitation with increased diarrheal disease, urban sanitation interventions have had mixed effects on health [6,13–19]. This variation in impact may be in part because interventions generally target the household only and do not address the entire sanitation chain [2,18,20]. In contrast, in rural areas, high community-level improved sanitation coverage has historically been required to achieve reductions in diarrheal disease, implying the need to reduce fecal contamination in both the private and public domains. However, the spatial heterogeneity of sanitation coverage has rarely been measured in rural or urban settings [3,21–23].

In urban areas, quantitative microbial risk assessments (QMRAs) have identified exposure to fecal contamination in the public domain, including open drains, as a high risk for children [4,5,24,25]. These exposures may result from poor containment of excreta associated with unsewered, onsite household sanitation (poor fecal sludge management, “FSM”) [7,26]. There has been little study of the effects of improved onsite sanitation, including improved pit latrines and ventilated improved pit (VIP) latrines with good pit-emptying services, on environmental fecal contamination in the public domain [27].

Although they may present some of the only practical solutions to improving sanitation access in dense, urban areas[28], there is mixed evidence about the effectiveness of shared sanitation, classified below “improved” on the sanitation ladder, at reducing fecal exposures. Shared sanitation includes any facility shared by multiple households that would otherwise, by design, be considered improved [9]. When compared to individual improved facilities, shared sanitation has been associated with increased prevalence of pediatric diarrhea; however, the causal mechanism by which it affects these health outcomes is unclear [29–31]. Both urban and rural studies have shown no consistent differences between shared and unshared sanitation when measuring fecal contamination within toilets, in stored household water, or on children’s hands [32,33]. The effect of shared sanitation on fecal contamination in public, urban environments, where it may be the only feasible and sustainable sanitation option, is being explored by ongoing studies [34–36].

Given the interconnectedness of public and private domains in cities, there is a need to understand whether household sanitation facilities—both individual and shared—reduce levels of fecal contamination in the public urban environment. This study examines whether the type and spatial heterogeneity of sanitation facilities are associated with fecal contamination, as measured by *E*. *coli* concentrations and enteric virus detection in soil and drain water, in the public domains of four low-income, urban neighborhoods of Accra, Ghana. In the SDG context, assessment of the type and coverage of onsite excreta containment will contribute to understanding the conditions under which sanitation coverage can lead to community-level benefits in dense, urban environments.

## Methods

### Study site

Data were collected as part of the SaniPath Study [24,37] in four low-income neighborhoods in Accra, Ghana between September 2011 and March 2013 in collaboration with the Water Research Institute of the Center for Scientific and Industrial Research Institute, Ghana (WRI), The Noguchi Memorial Institute for Medical Research of the University of Ghana (NMIMR), and the Training, Research, and Networking for Development (TREND) Group. The SaniPath Study was conducted to quantify the relative contributions of household- and neighborhood-level risks of exposure to fecal contamination through multiple environmental pathways.

Accra has two annual rainy seasons: March–July and September–October, with peak rainfall for 2012 during April–June and September–October. Soil and drain samples were collected from March–December 2012, and household surveys were conducted from August–September 2012. Public toilet surveys were conducted from March–September 2012. Though all were low-income areas, the four study neighborhoods (Alajo, Bukom, Old Fadama, and Shiabu) were selected for variation in types of settlements, location, flooding, demographic characteristics, and household and public sanitation coverage [24,25,37,38]. Further details on neighborhood selection and characteristics have been previously described [24,25,37,38].

### Ethics

All study protocols were approved by the Institutional Review Board (IRB) at Emory University and the NMIMR IRB, University of Ghana.

### Environmental sampling and processing

Samples of soil were collected in public places, and water in open public drains was collected at approximately 20 locations in each neighborhood indicated by community leaders and local field staff or observed as areas where children play or have contact with drains. Each location was sampled once during the study period. Global Positioning System (GPS) coordinates were collected at each location using a Garmin eTrex Venture HC device (Garmin Ltd., Olathe, KS, USA, accuracy of within 3 meters), and observations of the environmental characteristics of the sample location were noted. Samples were tested for *E*. *coli*, adenovirus, and genogroup I and II norovirus (GI and GII norovirus). *E*. *coli* was selected as an indicator of overall fecal contamination, while enteric adenovirus and norovirus were chosen as indicators of human-specific fecal contamination because of their high infection burden in recent studies of West African children [39–42]. Samples were tested for *E*. *coli* by membrane filtration using BBL MI agar (Becton Dickinson, Franklin Lakes, New Jersey, USA) following United States Environmental Protection Agency method 1604 [43]. For virus analyses, DNA and RNA extraction utilized the MP Bio FastSoil DNA kit (MP Bio, Santa Ana, CA, USA) and Qiagen viral RNA mini kit (Qiagen Sciences, Germantown, Maryland, USA), respectively. Samples were tested for enteric adenovirus and GI and GII norovirus by quantitative PCR or RT-PCR using published methods [44,45]. The QuantiFast Pathogen PCR or RT-PCR kit (Qiagen Sciences, Germantown, Maryland, USA) was used as a screening PCR for target viruses and assay inhibition. Positive samples and samples with inhibitors were quantified with the OneStep RT-PCR kit (Qiagen Sciences, Germantown, Maryland, USA). Further details about sample collection and processing are in S1 File and the SaniPath website [46].

### Household surveys

Within study neighborhoods, households were defined as a person or group of people sharing cooking or living areas. Compounds consisted of a group of households sharing the same structure. Surveys were conducted in 200 households per neighborhood, as defined by the criteria for a separate study to design a rapid assessment tool[37], and selected by dividing the neighborhood into four segments, randomly choosing a starting household within each segment, and conducting systematic sampling, as previously described [38]. GPS coordinates were collected at each household. The target respondent was the primary caregiver of the youngest child, generally the female head of household. The number of people living in the household and compound and the household’s ownership of animals were recorded.

Enumerators categorized the type of household sanitation facilities present by observation and classified them—based on JMP structural guidelines [9,38]—into either: 1) “contained” facilities (ventilated improved pit (VIP) or Kumasi ventilated improved pit (KVIP) latrines, pour-flush/flush toilets into a septic/sewage system, or traditional pit latrines with slabs); 2) “uncontained” facilities (bucket/pan latrines or other latrines); or 3) those with no facility present. Because few study households had uncontained, onsite facilities (56/265, 21%) with no significant spatial clusters observed, the “uncontained facilities” group was combined with the “no facilities” group for analysis. “Improved” or “unimproved” sanitation categories were not used because most facilities were shared by at least two households [9].

### Public toilet surveys

Surveys and observations at public toilets have been described previously [38]. GPS points were collected at all public toilets in a neighborhood during transect walks with a community leader, and a subset of those toilets in each neighborhood were observed and surveyed. Public toilets were classified into “contained” and “uncontained” categories, as described previously.

### Analyses

**Fig 1:**
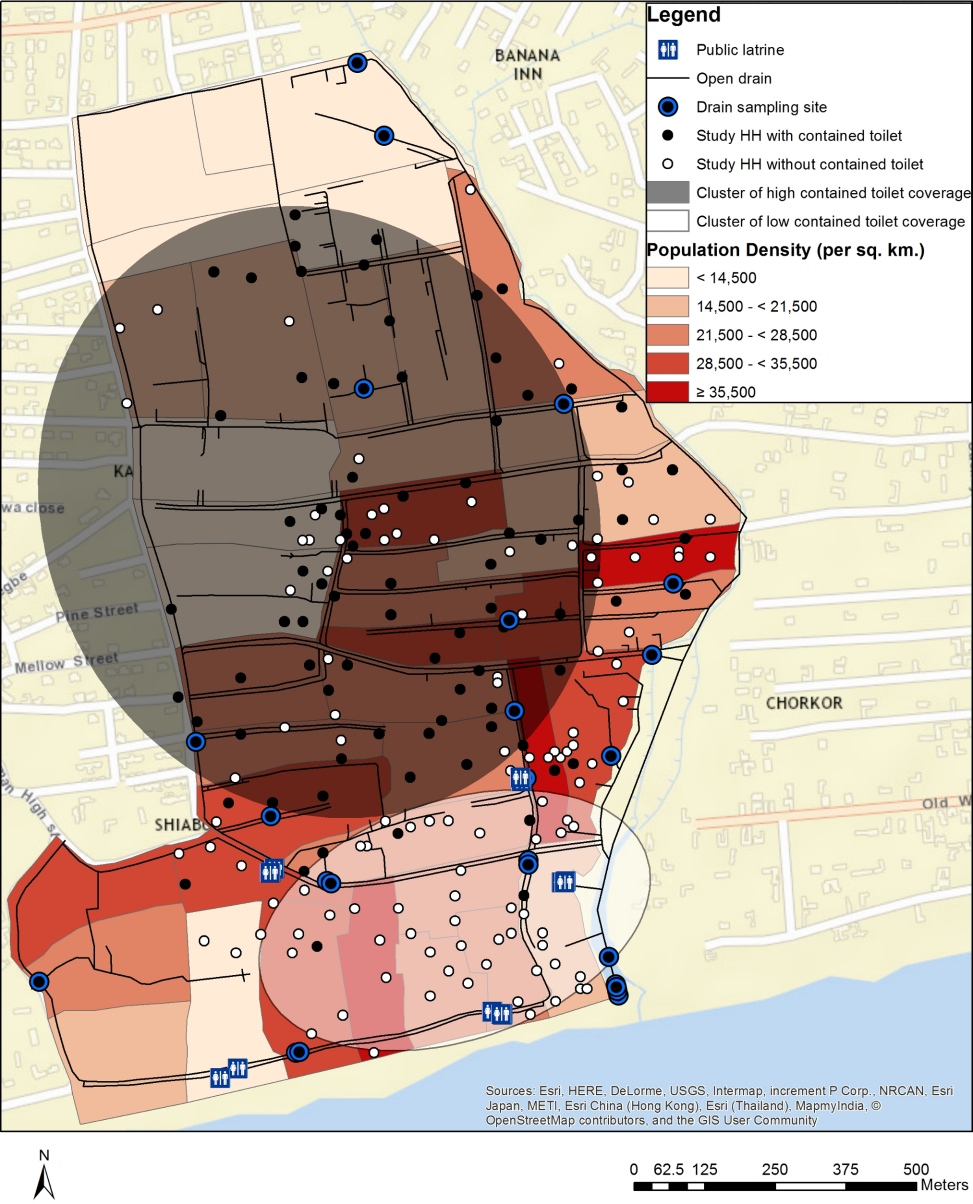
Neighborhood sanitation coverage and sample sites, Shiabu, Accra, Ghana. Drain sampling sites are illustrated using outlined circles. Households with a contained toilet are illustrated using black dots, while those without contained toilets (with uncontained toilets or no household sanitation facility present) are illustrated using white dots. Clusters of high (gray) and low (white) coverage of contained toilets are illustrated using ellipses. Although ellipses are drawn to estimate the locations of high- and low-coverage clusters, based on households inside/outside of the cluster, SaTScan software uses shape-based methodology (e.g. ellipses, circles) to scan the study area. Thus, the ellipse shape was selected *a priori* to allow for improved ability to scan irregularly-shaped areas (study neighborhoods). The software then scanned the study neighborhoods using ellipses of different sizes (up to 50% of the neighborhood’s households) to determine areas of significantly higher or lower coverage of the selected variable (any household sanitation, contained household sanitation) compared to the average of households not in that particular scan area (cluster) [48].

Population density surrounding environmental sampling locations was based on the 2010 Ghana Census data (Ghana Statistical Service, Accra, Ghana, estimated by Weeks et al. [47]). Samples were assigned the population density of the enumeration area where they were located (example: Fig 1).

Presence and type of household sanitation were evaluated for most-likely local spatial clustering within neighborhoods in SaTScan version 9.4 using Kulldorff’s Bernoulli spatial scan. The software evaluates binary outcomes in point data distributed in space to assess the degree of nonrandom clustering of ‘0’ or ‘1’ values [48]. The ellipse shape was selected for determining clusters because it provides an improved ability to scan irregularly-shaped areas, such as the study neighborhoods. Statistically significant (p < 0.05, as compared to the aspatial average of all households not in the cluster) clusters of high or low coverage were calculated in the software for the following variables: a) household sanitation presence vs. absence; and b) contained household sanitation presence vs. absence. For example, a spatial cluster of high coverage of contained sanitation was a group of households where the proportion of contained sanitation facilities was significantly higher than the proportion of contained sanitation facilities in the rest of that neighborhood (example: Fig 1).

Associations between sanitation and microbiological results for drain or soil samples were evaluated in two ways: a) by the presence or absence of a public toilet within 50 or 100m of the location; and b) by the presence of a spatial cluster of household sanitation—as defined above—within 50 or 100m of the location. For b), a sample was “within the cluster” if one or more households within the given distance (50 or 100m) of the sampling location were included in the spatial cluster. A maximum of one high cluster and one low cluster were detected per neighborhood, and clustering analysis did not allow clusters to overlap. Distances of 50m and 100m were chosen as a realistic scale for environment-household sanitation interactions in low-income urban neighborhoods, per previous urban sanitation assessments [49–51].

Statistical analyses were conducted in R version 3.1.2 (R Foundation for Statistical Computing, Vienna, Austria) using standard packages and the “logistf” package for penalized likelihood estimation [52]. Linear regression was used to assess continuous outcomes (*E*. *coli* concentrations), while logistic regression was used to assess binary outcomes (viral presence/absence). Regression models tested the associations between a sample being in a cluster and its fecal contamination levels by comparing samples collected within a certain type of cluster (e.g. a cluster of high coverage of contained sanitation) against all samples collected elsewhere in the neighborhood. Penalized likelihood estimation, referred to as Firth approximation, was used when separation—perfect or near-perfect estimation of an outcome by a single model parameter—was observed in logistic regression models [53]. The goal of regression modeling was to compare sub-neighborhood associations between sanitation and public domain fecal contamination, thus all regression models were adjusted for neighborhood (to account for differences in neighborhood-level characteristics) and population density (to adjust for total population in the area, given that a sample, and not a census, of households was conducted in study neighborhoods) around the sampling location. Other hypothesized covariates—including season of sample collection, presence of shade/sunlight at sampling location (for soil samples), presence of visible feces within 3m of the sampling location, presence of a sanitation facility within 30m of the sampling location and ownership of animals by households—were included in the final models if they were significant in the models adjusted for neighborhood. Effect modification of sanitation by population density was included in the model if significant at the 0.10 level, given the sub-aim to explore effect modification by population density on sanitation and the small dataset analyzed. An α of 0.05 was used for all other tests of significance. All data used in the analysis can be found in S2 File.

## Results

### Neighborhood demographics and coverage/clustering of household sanitation

Neighborhood demographics and sanitation coverage were compared across neighborhoods using chi-square tests or analysis of variance (ANOVA), and tested for spatial clustering (example: Fig 1). Sanitation coverage varied significantly between neighborhoods, with significant spatial heterogeneity at the sub-neighborhood level (Table 1). All public toilets observed in study neighborhoods were classified as contained, with the exception of those in Old Fadama. Because of the relatively low proportion of public toilets observed (43%) and the absence of within-neighborhood variation in contained/uncontained status, public toilets were further analyzed by presence/absence only. About 25% of households owned animals, though this attribute varied by neighborhood. Chickens (16.6% of households), cats (6.9%), goats (4.2%), and dogs (4.0%) were most commonly present. Household sanitation coverage was highest in Alajo (58.5%) and lowest in Old Fadama (1.5%); however, significant clusters of high coverage of any household sanitation and of contained household sanitation were detected in Bukom (79% coverage of any household sanitation, 44% coverage of contained household sanitation) and Shiabu (70% coverage of any household sanitation, 68% coverage of contained household sanitation). Household sanitation facilities were frequently shared between households (mean of almost 4 per facility) and people (mean of almost 11 per facility), and only 7% of all households surveyed had onsite, unshared (individual household) facilities. With the exception of the number of households sharing sanitation facilities, all other attributes varied significantly across neighborhoods, including population density, public toilet presence/absence, household ownership of domestic animals, prevalence of any—as well as contained—household sanitation facilities, and number of people sharing a household sanitation facility.

**Table 1:** Population density, animal ownership, and neighborhood sanitation, including spatial clustering^a^.

	Alajo	Bukom	Old Fadama	Shiabu	Overall	p-value^b^
	(n = 200)	(n = 199)	(n = 197)	(n = 197)	(n = 793)	
Population density (per km2)	21,475	75,927	50,835	21,594	42,458	<0.001
Total number of public toilets (number observed)	7 (4)	7 (5)	19 (5)	13 (6)	46 (20)	0.132
Number of contained public toilets (% of observed toilets^c^)	4 (100)	5 (100)	0 (0)	6 (100)	15 (75)	<0.001
HHs reporting owning domestic animals (%)	65 (32.5)	41 (20.6)	28 (14.2)	69 (35.0)	203 (25.6)	<0.001
HHs reporting disposing of children’s feces into drains (%)	5 (2.5)	1 (0.5)	11 (5.6)	7 (3.6)	56 (7.1)	0.028
# of HHs with ≥ 1 Sanitation Facility (%)	117 (58.5)	53 (26.6)	3 (1.5)	92 (46.7)	265 (33.4)	<0.001
# of HHs in High Coverage Cluster (% coverage^d^)	-	29 (79.3)	-	94 (70.2)		
# of HHs in Low Coverage Cluster (% coverage^d^)	10 (0.0)	66 (6.1)	-	42 (7.1)		
# of HHs with ≥ 1 Contained Sanitation Facility^e^ (%)	111 (55.5)	12 (6.0)	3 (1.5)	83 (42.1)	209 (26.4)	<0.001
# of HHs in High Coverage Cluster (% coverage^d^)	-	16 (43.8)	-	93 (67.7)		
# of HHs in Low Coverage Cluster (% coverage^d^)	-	-	-	55 (9.1)		
Sharing of household sanitation facilities^f^						
# of HHs with an unshared facility (%)	8 (4.0)	41 (20.6)	0 (0)	5 (2.5)	54 (6.8)	<0.001
Avg. # of HHs sharing a facility (SD)	3.7 (4.5)	2.6 (4.0)	3.0 (1.0)	4.3 (4.4)	3.9 (4.4)	0.946
Avg. # of people sharing a facility (SD)	11.4 (11.1)	4.5 (8.6)	5.8 (4.6)	12.5 (8.6)	10.7 (10.0)	0.039

^a^ “-” indicates no cluster was significant at the 0.10 level by Kulldorff’s Bernoulli spatial scan

^b^p-value for analysis of variance (ANOVA) for continuous variables, and chi-square test of independence for binary variables, across neighborhoods

^c^Reference for public toilet observations can be found here[38]

^d^Coverage of the attribute (e.g. household with any sanitation facility) in the cluster: number of households (% of households in cluster) with that attribute. All clusters presented were detected by Kulldorff’s Bernoulli spatial scan as significant at α = 0.05.

^e^A contained sanitation facility was one that, in the absence of sharing, would have been considered “improved” per the current JMP guidelines (including ventilated improved pit (VIP) or Kumasi ventilated improved pit (KVIP) latrine, pour-flush/flush toilets into a septic/sewage system, or traditional pit latrines with slabs);[9]

^f^Among households with at least one sanitation facility.

### Variation in environmental fecal contamination between urban neighborhoods and by season

To understand differences in environmental fecal contamination between neighborhoods, concentrations of *E*. *coli* and detection of enteric viruses (adenovirus and GI and GII norovirus) in soil and open drain samples from public areas were compared by ANOVA and chi-square tests of independence (Table 2). Across samples and organisms tested, no single neighborhood consistently had the highest concentrations or prevalence of positive detections. *E*. *coli* concentrations in soil varied significantly by neighborhood (p = 0.04), while concentrations in drains varied by neighborhood, but were not statistically significantly different (p = 0.053, Table 2A). *E*. *coli* concentrations in drain samples were high in all neighborhoods, varying by 0.3–0.8 log_10_CFU/100mL (Table 2A). GII norovirus detection in drains, but not GI norovirus or adenovirus detections in drains, varied significantly by neighborhood (Table 2B). *E*. *coli* concentration in soil was 2.4 log10 CFU/g overall and varied by 0.2–1.5 log_10_CFU/g across neighborhoods. Enteric adenovirus, GI norovirus, and GII norovirus were detected in 3 (4.4%), 1 (1.4%), and 0 (0%) soil samples, respectively, and thus were excluded from further analyses.

**Table 2:** *E*. *coli* concentrations and enteric virus detection in public domain samples, by neighborhood^a^.

	Alajo		Bukom		Old Fadama		Shiabu		Overall		p-value^b^
a) *E*. *coli* concentration	N	Mean (SD)	N	Mean (SD)	N	Mean (SD)	N	Mean (SD)	N	Mean (SD)	
Soil (log_10_CFU/g)	22	1.8 (1.6)	13	2.0 (1.5)	23	3.3 (1.5)	14	2.2 (1.2)	72	2.4 (1.6)	0.037
Drains (log_10_CFU/100mL)	26	8.4 (0.8)	19	8.9 (1.0)	23	8.1 (0.9)	25	8.8 (1.3)	90	8.5 (1.1)	0.053
b) Viral detection in drains^c^	N	Virus+ (%)	N	Virus+ (%)	N	Virus+ (%)	N	Virus+ (%)	N	Virus+ (%)	
Adenovirus	25	17 (68)	19	16 (84)	21	16 (76)	23	21 (91)	88	70 (80)	0.221
GI norovirus	25	4 (16)	19	5 (26)	19	6 (32)	21	6 (29)	84	21 (25)	0.642
GII norovirus	23	6 (26)	18	8 (44)	19	13 (68)	24	13 (54)	84	40 (48)	0.045

^a^Samples collected Mar.–Nov. 2012.

^b^The p-values were derived using ANOVA for continuous variables (*E*. *coli* concentrations), and chi-square test of independence for binary variables (viral detection), across neighborhoods

^c^Drain samples only. Viruses were detected in less than 5% of soil samples, thus soil sample results are presented in the text only.

To understand seasonal variation in environmental fecal contamination, *E*. *coli* concentrations (S1 Table) and enteric virus detection (S2 Table) were compared between rainy and dry seasons using linear and logistic regression, respectively, controlling for neighborhood. *E*. *coli* concentrations in both soil and drain samples did not vary significantly by season (S1 Table). GII norovirus was significantly less likely to be detected in drain samples in the September-October rainy season, compared to the dry season (odds ratio: 0.13, 95% confidence interval: 0.02–0.65); however, detection of other enteric viruses in drains did not differ significantly by season (S2 Table). Thus, season was included in all further models of GII norovirus, but not other organisms.

### Variation in environmental fecal contamination by animal ownership and population density

Associations between animal ownership and environmental fecal contamination were examined by linear regression models for *E*. *coli* concentrations in soil or drain samples and by logistic regression models for enteric viruses in drain samples, controlling for neighborhood. Household animal ownership was quantified within 50 or 100m of a sampling location as the number of study households owning an animal divided by the total number of study households within that radius. *E*. *coli* concentrations (S1 Table) and enteric virus detection (S2 Table) were not significantly associated with the prevalence of animal ownership within the given radii.

Associations between estimated population density and environmental fecal contamination were evaluated by linear and logistic regression, controlling for neighborhood. *E*. *coli* concentrations (S1 Table) and enteric virus detection (S2 Table) were not significantly associated with population density.

### Variation in E. coli contamination in soil by neighborhood sanitation

Associations between neighborhood sanitation, including presence of a public toilet and clustering of household sanitation coverage, and *E*. *coli* concentrations in soil were assessed by linear regression, controlling for neighborhood and population density. Almost all sampling locations (70/72) were exposed to sunlight, and the presence of visible feces (within 3m) or a toilet/open defecation area (within 30m) were not significantly associated with *E*. *coli* concentrations when adjusted for neighborhood (S1 Table), thus these variables were excluded from subsequent models. *E*. *coli* concentrations in soil samples were not significantly associated with the presence of a public toilet (Table 3). Compared to *E*. *coli* concentrations in soil samples from the rest of the study neighborhoods, *E*. *coli* concentrations in soil within 50m of spatial clusters of high coverage of any, or of contained, household sanitation were 1.3–1.8 log_10_CFU/g higher, though these differences were not statistically significant. Population density appeared to modify the association between clusters of low coverage of contained household sanitation and *E*. *coli* concentrations in soil: *E*. *coli* concentrations in soil within 50m of spatial clusters of low coverage of contained sanitation increased with increasing population density around the sampling location (p = 0.080).

**Table 3:** *E*. *coli* concentrations in soil in the public domain by sanitation coverage cluster.

Main effect of model^a^	Within 50m of soil sample (n = 58)			Within 100m of soil sample (n = 67)		
	β^b^	SE(β)	p-value^c^	β^b^	SE(β)	p-value^c^
Public latrine present	-0.23	0.48	0.622	0.06	0.41	0.884
Any HH sanitation						
High Coverage Cluster	1.29	1.58	0.418	-0.56	0.88	0.528
___Low Coverage Cluster	-0.33	0.76	0.661	-0.40	0.63	0.522
Contained HH sanitation						
High Coverage Cluster	1.84	1.10	0.100	1.01	0.92	0.278
Low Coverage Cluster	-1.25	0.92	0.179	-0.54	0.61	0.387
Low Coverage Cluster*Population density^d^	0.12	6.67 x 10^−2^	0.080			

^a^Linear regression models presented with estimates (β) and their standard errors (SE). All models are adjusted for neighborhood, population density around the location of the sample. Effect modification of the main sanitation variable by population density was tested in each model and included if p < 0.10 for the interaction term.

^b^Units are log_10_CFU/g.

^c^p-value for main effect of model.

^d^Per 10,000 per square kilometer.

### Variation in E. coli contamination in drain water by neighborhood sanitation

Associations between neighborhood sanitation and *E*. *coli* concentrations in drains were assessed using linear regression, controlling for neighborhood and population density. *E*. *coli* concentrations in drain water samples were significantly higher within 100m of public latrines, when compared to the rest of the neighborhood (p = 0.014, Table 4). Interestingly, the association between the presence of a public latrine and *E*. coli concentrations in drains was modified by population density (p = 0.088): increasing population density near a public latrine was associated with lower *E*. *coli* concentrations. In a separate model, *E*. *coli* concentrations in drain water samples within clusters of high coverage of contained household sanitation (using a 50m cutoff) were 3.66 log_10_CFU/100mL lower than in the rest of the study area (p = 0.008). This model also showed significant effect modification by population density on clusters of sanitation coverage: increasing population density within a cluster of contained household sanitation was significantly associated with higher *E*. *coli* concentrations in drains (p = 0.008). Compared to other sampling locations in the study area, *E*. *coli* concentrations were 1.29–3.85 log_10_CFU/100mL higher in drain water samples within clusters of low coverage of contained household sanitation (using 50m and 100m cutoffs). Though not always statistically significant, lower *E*. *coli* concentrations were generally associated with drains in or near clusters of high coverage of household sanitation facilities, while higher *E*. *coli* concentrations were generally associated with drains in or near clusters of low coverage of household sanitation facilities.

**Table 4:** *E*. *coli* concentrations in public drains by sanitation coverage cluster.

Main effect of model^a^	Within 50m of drain sample (n = 58)			Within 100m of drain sample (n = 72)		
	β^b^	SE(β)	p-value	β^b^	SE(β)	p-value
Public latrine present	0.26	0.35	0.449	0.91	0.36	0.014
Public latrine x Population density^c^				-7.62 x 10^−2^	4.39 x 10^−2^	0.088
Any HH sanitation						
High Coverage Cluster	-0.63	0.47	0.185	-0.63	0.41	0.132
___Low Coverage Cluster	0.13	0.36	0.710	0.28	0.31	0.371
Contained HH sanitation						
High Coverage Cluster	-3.66	1.32	0.008	-0.25	0.39	0.512
High Coverage Cluster x Population density^c^	0.93	0.34	0.008			
___Low Coverage Cluster	1.29	0.52	0.017	3.85	1.54	0.015
___Low Coverage Cluster x Population density^c^				-1.05	0.55	0.059

^a^Linear regression models presented with estimates (β) and their standard errors (SE). All models are adjusted for neighborhood and population density around the location of the sample. Effect modification of the main sanitation variable by population density was tested in each model and included if p < 0.10 for the interaction term.

^b^Units are log_10_CFU/100mL.

^c^Per 10,000 per square kilometer.

### Variation in enteric virus detection in drain water by neighborhood sanitation

Associations between neighborhood sanitation and adenovirus or norovirus detection in drains were assessed using logistic regression, controlling for neighborhood and population density. Models for GII norovirus also controlled for season, given previous results. Detection of viruses in drains was not significantly associated with public toilet presence or with clustering of household sanitation (S3 Table). Further, unlike in models for *E*. *coli* concentrations, no consistent trends in associations were observed.

## Discussion

This study examined associations between local coverage of sanitation infrastructure and fecal contamination in soils and drains in urban, public environments. *E*. *coli* concentrations in drain samples collected within spatial clusters of high coverage of contained household sanitation, using a 50m cutoff, were significantly lower than concentrations in samples from drains that were not in those clusters. *E*. *coli* concentrations in drains were generally a) lower in and around clusters of *high* coverage of sanitation facilities, and b) higher in and around clusters of *low* coverage sanitation facilities, especially contained household sanitation; however, these associations were not always statistically significant. Importantly, these trends in associations between environmental *E*. *coli* concentrations in drains and contained household sanitation infrastructure were present despite most households sharing sanitation facilities. *E*. *coli* concentrations in soil were not significantly associated with clustering of household sanitation, suggesting other, unmeasured factors may be important for future investigation. Detection of enteric viruses in drains also did not vary significantly with clustering of sanitation, suggesting different associations from those between sanitation and *E*. *coli*. Finally, population density did not have a clear directional association in its effect modification of the association between sanitation clusters and *E*. *coli* concentrations in soil and drains, suggesting that its potential effects may require further study.

This study is one of the first to examine associations between sanitation coverage—by type and spatial heterogeneity—and fecal contamination in the public environment, an important intermediate outcome related to downstream personal exposures and health outcomes in the F diagram [3]. Other studies that have evaluated the associations between household sanitation and fecal contamination have focused on the user’s immediate environment [32,33], which fails to account for potentially important exposures in the public domain [2]. Studies of fecal contamination in the public environment have examined drains at larger (neighborhood or city) scales in sub-Saharan Africa and southeast Asia and observed high levels of fecal contamination in drains (though *E*. *coli* concentrations in this study were higher). However, these studies did not link this contamination to community- or neighborhood-levels, spatial heterogeneity, or types of sanitation coverage [5,54–61].

Associations between clusters of high coverage of contained household sanitation and lower *E*. *coli* concentrations in drains can be explained by both functional containment of human excreta at the household and relatively high local coverage of sanitation (44–68%) surrounding the households and sampling sites [21–23]. Containment of human excreta is the primary role of sanitation within the environment [3]. Open drains are a common fate for human excreta from uncontained household sanitation facilities in low-income urban areas, and most excreta in drains remains untreated, presenting a high risk fecal exposure pathway [4,5,25,26,62]. The effectiveness of containment may moderate the association between household sanitation coverage and concentrations of *E*. *coli* in the environment, as observed in this study. *E*. *coli* concentrations in drains showed consistent trends (lower concentrations in or near clusters of high coverage of household sanitation facilities, and vice versa) for both any and contained sanitation facilities. However, only clusters of high coverage of contained sanitation facilities—those that contain excreta onsite, per JMP guidelines [9]—had *significantly* lower *E*. *coli* concentrations in drains. Compared to sanitation facilities that did not contain excreta onsite or households without sanitation facilities at all, these contained household sanitation facilities keep fecal contamination away from locations of potential human contact, such as drains [9,26].

Most household sanitation facilities were considered “contained” (209/265, 79%) and thus did not routinely discharge directly into drains. We hypothesize that the primary ways that *E*. *coli* were entering the drains were through direct discharge from the non-contained toilets (as well as those not enumerated by the study) into drains, and to a lesser extent, open defecation by households without access to sanitation facilities. In addition, occasional overflow of household toilets or pit latrines into drains, run-off due to rainfall and flooding, and deliberate disposal of children’s feces into drains could have also introduced high concentrations of *E*. *coli* into drains. Because the 21% of households with uncontained facilities likely contributed to fecal contamination in drains, future studies should focus on fecal contamination surrounding households with “contained” vs. households with “uncontained” facilities, since we lacked sufficient sample size to examine the latter group specifically in this study. Children’s feces were infrequently reported to be emptied into drains (1–6% by neighborhood) and were mostly disposed of in rubbish, but much rubbish was observed in the open drains. The Ghana Statistical Service has estimated that up to 74% of liquid waste across Accra may be discharged into drains, suggesting improper disposal—whether due to engineered infrastructure, behaviors, or both—as an important contributor to fecal contamination in the public domain [63]. Emptying onsite containment system is necessary to provide safe, sustainable sanitation in urban areas, and poor emptying practices can pose a risk of environmental contamination. However, information on local septic tank and pit latrine emptying practices was not collected in this study. Further research should build on this work by examining the entire sanitation chain. Critically, the drains in our study neighborhoods in Accra were connected to those of other neighborhoods where fecal contamination was not measured, and this could have affected the *E*. *coli* concentrations detected at our sampling locations.

Associations between clusters of high coverage of household sanitation and lower *E*. *coli* concentrations in drain water were observed even though most household toilets were shared. Shared sanitation is common in Accra, and studies of shared sanitation—when compared to individual household toilets—have observed elevated prevalence and odds of diarrhea, resulting in the exclusion of shared sanitation from definitions of improved sanitation [9,28,30,38,64]. Previous research on the associations between fecal contamination and shared sanitation has focused on within-toilet maintenance and contamination. Our results suggest that containment of excreta by shared sanitation facilities reduced concentrations of fecal contamination in the nearby public environment despite sharing by almost 4 households or 11 people, on average. However, the relationship between shared sanitation and fecal contamination in the public domain should be explored further in larger studies that specifically target shared sanitation[36].

Though *E*. *coli* concentrations in drains in spatial clusters of high coverage of contained household sanitation were significantly lower than the rest of the study area, all *E*. *coli* concentrations detected suggest fecal contamination levels that were high enough to pose significant health risks to children upon contact [25]. Notably, *E*. *coli* concentrations in drains in the neighborhood with the lowest coverage of toilets (Old Fadama, 8.1 log_10_CFU/100mL, < 2% coverage of any or contained household sanitation facilities) were similar to those in the neighborhood with highest coverage (Alajo, 8.4 log_10_CFU/100mL, 56–59% coverage of any or contained household sanitation facilities), which suggests that contained sanitation coverage at the neighborhood level was not sufficiently high to reduce *E*. *coli* concentrations below that of raw feces. This finding is consistent with evidence from previous studies in rural areas that suggested a 75–80% coverage of household sanitation was necessary to achieve meaningful community-level changes in health[21–23]; however, the community-level coverage necessary to achieve reductions in environmental contamination has not been studied directly.

Coverage of household sanitation was not associated with *E*. *coli* concentrations in soil. In fact, nonsignificant trends indicated *E*. *coli* concentrations in soil may have been higher around clusters of high coverage of household sanitation facilities than other neighborhood locations, suggesting soil contamination may be influenced by other, unmeasured factors. Previous research in rural households has suggested that fecal contamination in soil varies with human foot traffic in the area, regardless of the type or coverage of local sanitation [65]. Although fecal contamination in soil may have been influenced by foot traffic and mixing of people, we did not observe an association between *E*. *coli* concentrations in soil and population density. Future studies should examine this hypothesis in urban settings and include measurement of soil moisture content, an important covariate for soil contamination, to allow standardization of soil *E*. *coli* concentrations per gram dry weight [65].

Detection of enteric viruses in drain water was not associated with coverage of household sanitation, which may indicate differences between *E*. *coli* concentrations and enteric virus presence in sewage, but may also reflect limitations of the laboratory methods for virus detection in these samples. Adenovirus has been used to indicate and track human fecal contamination in rural and aquatic environments: areas subject to less regular human activity than urban open drains [66,67]. Both human adenovirus and GII norovirus have frequently been detected in urban drain water, including drain water in Accra [61,66–71]. Thus, the lack of correlation between enteric viruses and sanitation coverage was unexpected. The use of presence/absence data, the high lower limits of detection for the RT-PCR assays, and the presence of PCR inhibitors in environmental media may have limited the precision of our analyses [72,73]. Though the viral results differed from *E*. *coli* results in this study, variation in environmental concentrations of enteric viruses should be evaluated further in sanitation studies, as they could provide persistent, human-specific indicators of fecal contamination [69].

Population density alone was not associated with fecal contamination outcomes and did not modify the associations between sanitation and fecal contamination in a consistent manner. While increasing population density was associated with significantly higher *E*. *coli* concentrations in drains in clusters of high coverage of contained sanitation facilities, it was also associated with lower *E*. *coli* concentrations in drains near public latrines. These results are similar to previous work on sanitation, population density, diarrhea, and enteric infections in Guatemala [74], which showed that population density was not, by itself, a significant predictor of enteric infection. Further, in that study, the authors also observed that population density was not a strong effect modifier of risk of enteric infection from poorer sanitation. While we did not measure enteric infection, our observation of significant modification of associations between household sanitation clusters and fecal contamination may run contrary to this previous work; however, we also had a smaller sample size than the previous study, which may affect our ability to generalize these conclusions further. Overall, further examination of the potential links between population density and sanitation-related fecal contamination and health outcomes is necessary, given recent demographic trends. Ongoing work on population density in the context of improving shared sanitation infrastructure [36] will provide valuable insights on these relationships.

Our findings utilize *E*. *coli* as an indicator of fecal contamination in environmental samples, which requires careful consideration of limitations. Detection of *E*. *coli* does not distinguish between human and animal sources [75,76], and *E*. *coli* may be detected year-round in some soils and waters in tropical climates [77], thus we cannot definitively attribute human excreta as the source. Although the *E*. *coli* concentrations measured in this study did not vary significantly with animal ownership in nearby study households, suggesting human feces may have been the primary source of the *E*. *coli* detected, this may have also been due to relatively low household ownership of animals (26%) and other unmeasured factors—including the presence of animals in the area due to non-study households [76]. Animals and associated fecal contamination represent an important, established source of fecal pathogens and a significant risk factor for human health [78] that were not measured in this study.

*E*. *coli* concentrations in environmental samples may also vary seasonally, especially with rainfall [77,79]. While season was not observed to be a significant predictor of the *E*. *coli* concentrations measured in this study and was not a significant confounder, rainfall was not directly measured during the sampling period and data for retrospective rainfall was unavailable, restricting modeling of seasonal variation to more crude approximations by month.

The limited environmental sample size, combined with the purposive sampling technique, should be considered when contextualizing these results with those of other studies. Sample size restricted the number and types of covariates that could be adjusted for in the analytical models, and may have limited overall power to detect statistically significant associations, given the observed variation in *E*. *coli* concentrations. Although the sampling locations were selected independently of local sanitation coverage, future studies should conduct random sampling to improve the generalizability of results. The sampling technique also limited our ability to measure and resolve sanitation clustering, though this was mitigated by using systematic sampling to estimate the underlying household distribution in space and by using a spatial scan statistic robust to uneven population distributions [80]. Use of spatially-explicit census data also improved modeling of sub-neighborhood spatial heterogeneity, and our estimated sanitation coverage for these study neighborhoods approximated that of census-level data collected by the Ghana Statistical Service/Cooperative Housing Foundation/Accra Metropolitan Assembly in 2010 [81]. Observation of household sanitation facilities, rather than self-report, was also a study strength that limited response bias.

Given the importance of associations between exposure to fecal contamination in the public environment and pediatric enteric infection, these findings provide new evidence that high local coverage of contained household sanitation, including shared sanitation, may reduce environmental fecal contamination in a high risk pathway near the household.[4,5,40–42,82,83] This finding underscores the importance of further research that measures the effects of household-level sanitation and FSM on environmental fecal contamination, and subsequent health risk, in the public domain. Future studies should estimate risk from environmental fecal contamination throughout the sanitation chain—specifically including the fate and transport of fecal sludge in the environment, especially in the public domain—to develop effective FSM strategies.

## Supporting information

S1 File Supplemental methods information. (DOCX)

S2 File Analytic dataset. (XLS)

S1 Table *E*. *coli* concentrations in soil and drains by season, population density, and local household animal ownership. (DOCX)

S2 Table Enteric virus detection in drains by season, population density, and local household animal ownership. (DOCX)

S3 Table Adenovirus, NoV GI, and NoV GII detection in public drains by sanitation coverage cluster. (DOCX)
