# Supplementary material for: Urban sanitation coverage and environmental fecal contamination: Links between the household and public environments of Accra, Ghana
Source: PLoS One. 2018 Jul 3;13(7):e0199304. doi: 10.1371/journal.pone.0199304 (PMC6029754; doi:10.1371/journal.pone.0199304)
Supplement: S1 File — (DOCX) [file pone.0199304.s001.docx]

**S1 File**

**Soil:** Soil samples were collected using a sterile plastic scoop and sterile 250 mLWhirl-Pak bags (Nasco, Fort Atkinson, WI, USA). Seven separate samples totaling approximately 30g were collected within a 3m radius and combined into a single 250 mL Whirl-Pak bag. Samples were sealed, placed on ice in a cooler, and transported to WRI within 6 hours of collection. At the lab, the sample was weighed, mixed by rotation, and stored at 4°C until analysis. At the time of collection, the staff noted the date and whether the sample location was exposed to sunlight, within 3m of feces, and/or within 30m of a toilet or open defecation area.

Prior to membrane filtration, 10g of the composite sample were weighed into a sterile 50 mL conical tube with 20 mL of sterile phosphate-buffered saline (PBS). The sample was then vortexed for 30 seconds, adjusted to a pH of 9.0 by addition of 0.1N sodium hydroxide (NaOH), and shaken vigorously on a rotator or shaker for 30 minutes at room temperature. After 15 minutes of settling, 10 mL of the supernatant were aliquoted into a new sterile 50 mL conical tube, from which subsequent aliquots were taken for 1:10^0^, 1:10^1^, and 1:10^2^ dilutions for membrane filtration. In addition, 1.5 mL of undiluted sample supernatant were aliquoted for PCR analysis at NMIMR. For PCR testing of GI and GII norovirus, 0.18g polyethelene glycol (PEG) was added, followed by centrifugation for 20 minutes at 6000 RPM and resuspension in sterile water, to concentrate the virus for RNA isolation.

**Drain water:** Samples of drain water were collected using a sterile bailer or a Sludge Nabber (Nasco, Fort Atkinson, WI, USA) with sterile 500 mL Whirl-Pak bags. The bailer or Sludge Nabber was submerged (or turned horizontally in shallow water) until full. Samples were deposited into the 500 mL Whirl-Pak bag until filled. Whirl-Pak bags were sealed and labeled with the date, placed on ice in a cooler, and transported to WRI, where they were stored at 4°C until analysis.

Samples were diluted 1:10^5^, 1:10^6^, and 1:10^7^ in sterile PBS prior to membrane filtration. In addition, 1.5 mL of undiluted sample were aliquoted for PCR analysis at NMIMR. For PCR testing of GI and GII norovirus, 0.18g PEG was added, followed by centrifugation for 20 minutes at 6000 RPM and resuspension in sterile water, to concentrate the virus for RNA isolation.

All soil and drain samples were quantifiable above the lower limit of detection (1 CFU/100mL) for *E. coli* by membrane filtration. Samples evaluated by PCR were considered positive if both wells in OneStep analysis had cycle threshold (C_t_) values ≤ 41 and within 5 C_t_ of one another. Estimated lower limits of detection for adenovirus, GI norovirus, and GII norovirus were 6.7 x 10^2^, 3.3 x 10^4^, and 6.7 x 10^3^ genome copies, respectively, per gram for soil samples and 3.3 x 10^4^, 1.7 x 10^6^, and 3.3 x 10^5^ genome copies per 100mL, respectively, for drain water samples.
