# Supplementary material for: Urban sanitation coverage and environmental fecal contamination: Links between the household and public environments of Accra, Ghana
Source: PLoS One. 2018 Jul 3;13(7):e0199304. doi: 10.1371/journal.pone.0199304 (PMC6029754; doi:10.1371/journal.pone.0199304)
Supplement: S1 Table — (DOCX) [file pone.0199304.s003.docx]

### Table S1: E. coli concentrations in soil and drains by season, population density, and local household animal ownership

| Main effect of model^a,1^ | *E. coli* in soil sample^b^ | | *E. coli* in drain sample^c^ | |
| --- | --- | --- | --- | --- |
|  | β | SE(β) | β | SE(β) |
| Presence of visible feces (within 3m) | 0.05 | 0.65 | - | - |
|  |  |  |  |  |
| Presence of toilet/open defecation area (within 30m) | -0.69 | 0.39 | - | - |
|  |  |  |  |  |
| Rainy season (Mar-July)^d^ | -0.06 | 0.62 | 0.24 | 0.34 |
| Rainy season (Sept-Oct)^d^ | -0.73 | 0.61 | 0.03 | 0.37 |
|  |  |  |  |  |
| Population density^d,e^ | -1.29 x 10^-6^ | 3.61 x 10^-6^ | -7.98 x 10^-7^ | 2.01 x 10^-6^ |
|  |  |  |  |  |
| Prevalence of reported household animal ownership^d,f^  Within 50m  Within 100m | 2.10 x 10^-2^  8.38 x 10^-2^ | 6.46 x 10^-2^  9.46 x 10^-2^ | -2.52 x 10^-2^  -5.31 x 10^-2^ | 7.00 x 10^-2^  6.68 x 10^-2^ |

^a^Linear regression models presented with estimates (β) and their standard errors (SE). ^b^log_10_CFU/g; ^c^log_10_CFU/100mL; ^d^Adjusted for neighborhood.  ^e^Per person per km^2^; ^f^Estimates are per 10% increase in prevalence of reported animal ownership; ^1^No significant associations between *E. coli* concentrations and main effects were observed in any of these models. Data on the presence of visible feces and the presence of a toilet or open defecation area near the sample collection site were only collected for soil sampling locations.
