# Supplementary material for: Urban sanitation coverage and environmental fecal contamination: Links between the household and public environments of Accra, Ghana
Source: PLoS One. 2018 Jul 3;13(7):e0199304. doi: 10.1371/journal.pone.0199304 (PMC6029754; doi:10.1371/journal.pone.0199304)
Supplement: S2 Table — (DOCX) [file pone.0199304.s004.docx]

Table S2: Enteric virus detection in drains by season, population density, and local household animal ownership

| Main effect of model^a^ |  |  |  |
| --- | --- | --- | --- |
|  | Adenovirus  OR (95% CI) | GI norovirus  OR (95% CI) | GII norovirus  OR (95% CI) |
| Rainy season (Mar-July)^b^ | 0.11 (0.00, 1.04) | 0.65 (0.13, 3.78) | 0.23 (0.04, 1.01) |
| Rainy season (Sept-Oct)^b^ | 0.18 (0.00, 2.01) | 0.38 (0.05, 2.56) | 0.13 (0.02, 0.65)^†^ |
|  |  |  |  |
| Population density^b,c^ | 1.00 (0.99, 1.00) | 0.99 (0.99, 1.00) | 1.00 (0.99, 1.00) |
|  |  |  |  |
| Prevalence of reported household animal ownership^b,d^  Within 50m  Within 100m | 1.28 (0.90, 1.94)  1.13 (0.82, 1.61) | 1.19 (0.84, 1.72)  1.42 (0.97, 2.19) | 1.02 (0.76, 1.37)  0.92 (0.67, 1.25) |

^a^Logistic regression models presented, with odds ratio (OR) and 95% confidence interval (95% CI). ^b^Adjusted for neighborhood.  ^c^Per person per km^2^; ^d^Estimates are per 10% increase in prevalence of reported animal ownership within the given vicinity (with prevalence calculated as A/B, where A is the number of households surveyed within the given vicinity that reported having animals and B is the total number of households surveyed within the given vicinity); ^†^p < 0.05
