# Supplementary material for: Urban sanitation coverage and environmental fecal contamination: Links between the household and public environments of Accra, Ghana
Source: PLoS One. 2018 Jul 3;13(7):e0199304. doi: 10.1371/journal.pone.0199304 (PMC6029754; doi:10.1371/journal.pone.0199304)
Supplement: S3 Table — (DOCX) [file pone.0199304.s005.docx]

Table S3: Adenovirus, NoV GI, and NoV GII detection in public drains by sanitation coverage cluster*^a^*

| Main effect of model | 50m vicinity of drain sample (n = 58) | | | 100m vicinity of drain sample (n = 72) | | |
| --- | --- | --- | --- | --- | --- | --- |
|  | Adenovirus  OR (95% CI) | GI norovirus  OR (95% CI) | GII norovirus  OR (95% CI) | Adenovirus  OR (95% CI) | GI norovirus  OR (95% CI) | GII norovirus  OR (95% CI) |
| Public toilet presence | 0.35  (0.07, 1.70) | 0.15  (0.00, 1.31)^b^ | 1.12  (0.21, 6.54) | 0.46  (0.12, 1.74) | 0.30  (0.04, 1.30) | 0.56  (0.15, 2.00) |
| Any HH sanitation  High Coverage Cluster    Low Coverage Cluster | 0.79  (0.07, 18.4)^b^  2.70  (0.37, 55.7)^b^ | 0.18  (0.00, 2.09)^b^  0.40  (0.02, 2.94) | 0.25  (0.02, 2.72)  1.25  (0.20, 8.41) | 1.08  (0.10, 25.0)  1.88  (0.37, 14.7) | 0.32  (0.01, 2.80)  0.56  (0.07, 2.94) | 0.56  (0.06, 4.53)  1.56  (0.36, 6.89) |
| Contained HH sanitation^c^ | |  |  |  |  |  |
| High Coverage Cluster  Low Coverage Cluster | 4.18  (0.00, 578)^b^  0.20  (0.00, 3.13)^b^ | 0.51  (0.02, 4.53)  2.01  (0.14, 51.9) | 0.67  (0.06, 5.87)  0.87  (0.07, 9.87) | 6.17  (0.58, 846)^b^  0.20  (0.00, 3.03)^b^ | 1.98  (0.29, 13.7)  3.63  (0.34, 87.4) | 0.86  (0.13, 5.37)  1.19  (0.12, 11.6) |

^a^Logistic regression model presented with odds ratio (OR) and 95% confidence interval (95% CI), adjusted for neighborhood and population density around the location of the sample. GII norovirus models are also adjusted for season of sample collection; ^b^estimated by Firth approximation; ^c^ A contained sanitation facility was one that, in the absence of sharing, would have been considered “improved” per the current JMP guidelines (including ventilated improved pit (VIP) or Kumasi ventilated improved pit (KVIP) latrine, pour-flush/flush toilets into a septic/sewage system, or traditional pit latrines with slabs)
